# Combined Transcriptome and Metabolome Analysis of *Musa nana* Laur. Peel Treated With UV-C Reveals the Involvement of Key Metabolic Pathways

**DOI:** 10.3389/fgene.2021.792991

**Published:** 2022-01-27

**Authors:** Ming-zhong Chen, Xu-Mei Zhong, Hai-Sheng Lin, Xiao-Ming Qin

**Affiliations:** ^1^ College of Food Science and Technology, and Guangdong Provincial Key Laboratory of Aquatic Product Processing and Safety, Guangdong Ocean University, Zhanjiang, China; ^2^ Yangjiang Polytechnic, Yangjiang, China

**Keywords:** antioxidant activity, banana storage, flavonoid biosynthesis, hypersensitive response, postharvest treatment, UV-C treatment, phenylpropanoid biosynthesis

## Abstract

An increasing attention is being given to treat fruits with ultraviolet C (UV-C) irradiation to extend shelf-life, senescence, and protection from different diseases during storage. However, the detailed understanding of the pathways and key changes in gene expression and metabolite accumulation related to UV-C treatments are yet to be explored. This study is a first attempt to understand such changes in banana peel irradiated with UV-C. We treated *Musa nana* Laur. with 0.02 KJ/m^2^ UV-C irradiation for 0, 4, 8, 12, 15, and 18 days and studied the physiological and quality indicators. We found that UV-C treatment reduces weight loss and decay rate, while increased the accumulation of total phenols and flavonoids. Similarly, our results demonstrated that UV-C treatment increases the activity of defense and antioxidant system related enzymes. We observed that UV-C treatment for 8 days is beneficial for *M. nana* peels. The peels of *M. nana* treated with UV-C for 8 days were then subjected to combined transcriptome and metabolome analysis. In total, there were 425 and 38 differentially expressed genes and accumulated metabolites, respectively. We found that UV-C treatment increased the expression of genes in secondary metabolite biosynthesis related pathways. Concomitant changes in the metabolite accumulation were observed. Key pathways that were responsive to UV-C irradiation include flavonoid biosynthesis, phenylpropanoid bios6ynthesis, plant-pathogen interaction, MAPK signaling (plant), and plant hormone signal transduction pathway. We concluded that UV-C treatment imparts beneficial effects on banana peels by triggering defense responses against disease, inducing expression of flavonoid and alkaloid biosynthesis genes, and activating phytohormone and MAPK signaling pathways.

## Introduction

China is a major consumer as well as producer of banana. In 2019, China produced 11,655.65 thousand metric tons of bananas; Guangdong being the major banana production area (4,648.31 thousand metric tons) (https://www.statista.com/statistics/242954/banana-production-in-china-by-region/). Furthermore, China imported 1.94 million tons of banana in 2019 alone (www.producereport.com). The produced and imported banana needs to be transported to the consumers fresh and disease free. In order to keep bananas fresh, there are several storage methods; one of which is getting popularity nowadays i.e., treatment with ultraviolet C (UV-C) irradiation. UV-C suppresses several postharvest diseases. It is a nonionizing and nonthermal way which improves the storability of fruits and reduces deterioration of fruits and vegetables by triggering defense responses (Wituszyńska and Karpiński, 2013; Ding and Ling, 2014; Xu and Liu, 2017). However, the dose, application period, and the mechanism of UV-C action on fruits is still at the exploratory stage.

UV-C has been proven to be much safer than the longer wavelength UVs i.e., UV-A and UV-B. Also, UV-C is considered more effective in controlling bacteria than the longer wavelengths (Bintsis et al., 2000). In particular reference to banana, UV-C treatment (0.03 KJ/m^2^) has been reported to improve abiotic stress tolerance e.g., reduced chilling injury in peels. This increased tolerance was attributed to reduced damage to membranes, lower browning and damage to chlorophyll (Pongprasert et al., 2011). Another study tested the effect of 0.01–0.30 KJ/m^2^ UV-C irradiation on the control of crown rot disease, quality of postharvest fruits, and changes in antioxidant capacity of banana fruits (*Musa AAA* “Berangan”). The study found that UV-C irradiation was able to not only control the crown rot disease severity but also resulted in increase of total phenolics, ferric reducing antioxidant power (FRAP), and 1,1-diphenyl-2-picrylhydrazyl (DPPH) values (Mohamed et al., 2017). These studies indicate that the effect of UV-C treatment on banana (and other fruits) is dose dependent and can induce changes in antioxidant capacity, secondary metabolites, and disease resistances. However, the knowledge on the molecular mechanism of UV-C irradiation effects in these processes is still not explored in detail.

It has been demonstrated that UV-C treatments eliminate/control the postharvest diseases and improve shelf-life of fruits. At the same time, it does not impart negative physiological impacts on the stored tissues of the fruits and vegetables (Urban et al., 2016). In this regard, it is known that UV-C treatment may delay senescence and induce the biosynthesis of secondary metabolites, which have health benefits (Poiroux-Gonord et al., 2010). Though a complete understanding of the molecular control of such effects is unknown, studies reported that UV-C changes the expression of several genes (succinic dehydrogenases and cytochrome c oxidases) which control the important steps in tricarboxylic acid cycle (Yang et al., 2014). Similarly, how does UV-C treatment affect reactive oxygen species (ROS) generation and delay oxidative damage is not completely understood. However, it is known that when fruits (e.g., freshly harvested mangoes, strawberries, and pineapples) are treated with UV-C irradiation, the activity of enzymes related to antioxidant systems as well as defense mechanisms is enhanced (González Aguilar et al., 2007; Erkan et al., 2008; Alothman et al., 2009a). Regardless of such a diverse knowledge, there are limited studies which explored the gene expression of pathways that are associated with antioxidant systems, defense responses, and similar other pathways that could be associated with signaling and growth e.g., hormone signaling and MAPK signaling pathways (Urban et al., 2016). Since, the postharvest handling of different fruits differs, the mechanism underlying UV-C induced changes in the stored tissues could be different. Similarly, the tissue structure, biochemical nature, and genetic make-up of the fruits may differ, therefore, the UV-C dose, storage conditions, and shelf-life will need separate studies to explore related mechanisms.

Recent developments in transcriptome and metabolome profiling have enabled researchers to explore the key molecules that are expressed in response to specific stimuli. For example, metabolome profiling of mangosteen subjected to different ripening treatments enabled researchers to understand the accumulation of certain metabolites in response to the applied treatments (Parijadi et al., 2019). Another study used multi-omics (transcriptome and metabolome) approach to delineate the effects of blue LED on flavonoid and lipid metabolism in tea (Wang et al., 2020a). In a similar attempt, we irradiated *Musa nana* Laur. with 0.02 KJ/m^2^ UV-C for different days after harvesting (0, 4, 8, 12, 15, and 18 days) under similar storage conditions. Based on different physiological indicators, we selected the bananas irradiated for 8 days and studied the transcriptome and metabolome profiles of the peels. Through this approach, we report the key transcriptomic and metabolomic changes regarding flavonoid biosynthesis, phenylpropanoid biosynthesis, phytohormone signaling, and defense responses in *M. nana* peel under the influence of UV-C treatment.

## Results

### Physiological Analyses

#### Effect of UV-C Treatments on Banana Peel Appearance and Quality

**FIGURE 1:**
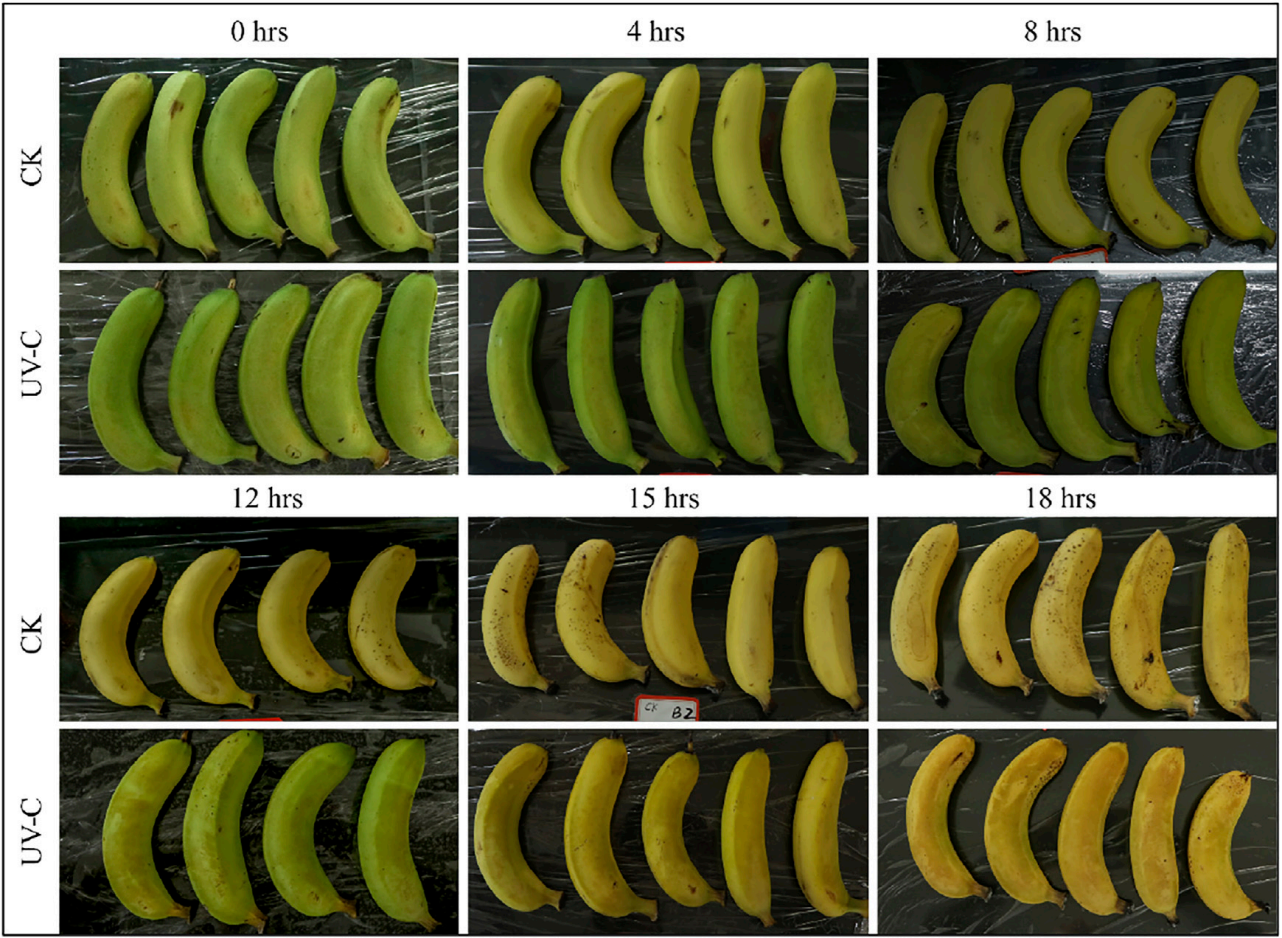
*M. nana* peel treated with UV-C (0.02 kJ/m^2^) or without UV-C (CK) for 0, 4, 8, 12, 15, and 18 days.

**FIGURE 2:**
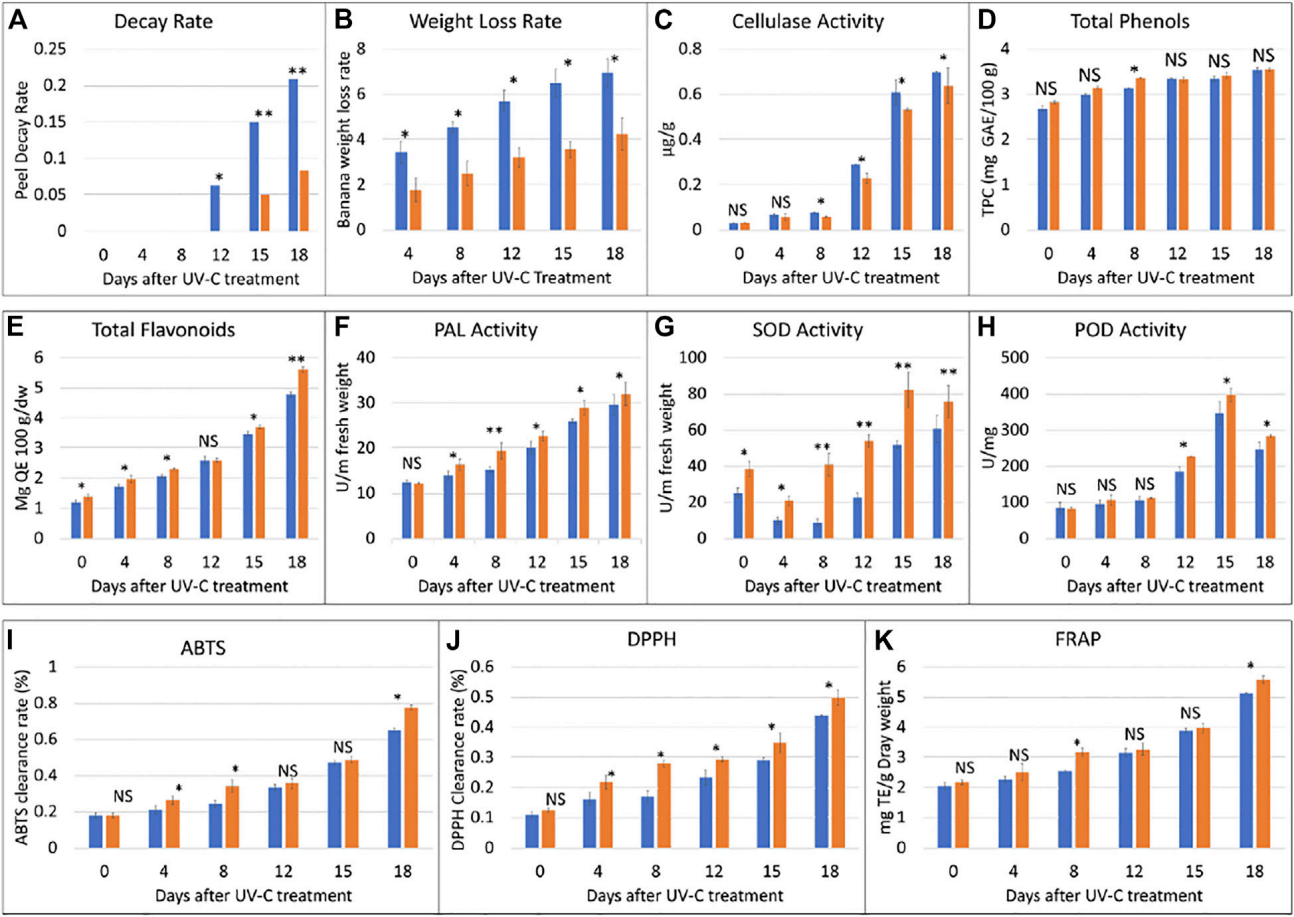
Physiological index analyses of banana peel treated with UV-C (0.02 kJ/m^2^) or without UV-C (CK) for 0, 4, 8, 12, 15, and 18 days. **(A)** peel decay rate, **(B)** weight loss rate, **(C)** cellulase activity, **(D)** total phenols, **(E)** total flavonoids, **(F)** phenylalanine ammonia lyase (PAL) activity, **(G)** superoxidase dismutase (SOD) activity, **(H)** peroxidase (POD) activity, **(I)** 2, 20 -azinobis-(3-ethylbenzothiazoline-6-sulfonic acid) (ABTS), **(J)** 1,1-diphenyl-2-picrylhydrazyl radical scavenging capacity (DPPH), and **(K)** ferric reducing antioxidant power (FRAP). The error bars represent standard deviation. The ns, *, and ** show that the differences between UV-C and CK treatments are non-significant, significant, and highly significant, respectively. The blue and orange bars represent CK and UV-C treated banana samples.

In this study, *M. nana* peel was irradiated with a dose of 0.02 KJ/m^2^ for 0–18 days under controlled temperature and humidity. We observed that, when banana was stored under the UV-C dose for a longer time i.e., 18 days, a few black spots appeared and the peel was shiny, while the CK peel had more black spots and its color was light yellow (Figure 1). There was no decay from 0 to 8 days, while from day 12–18, the decay rate of the UV-C group showed upward trend, while that of CK group was highly significantly low (*p* < 0.01) (Figure 2A). The weight loss rate of the CK was higher (significant at *p* < 0.01) than UV-C group at all time points (Figure 2B). These two observations suggest that UV-C treatment up to 8 days controls the banana peel decay rate, while the weight loss rate remains lower for UV-C treated bananas. Another indicator of fruit peel quality is the activity of cellulase activity, where it is known that its activity affects the hardness and the quality of fruits and vegetables (Kannahi and Elangeswari, 2015). We observed an increased cellulase activity with an increase in time. The CK cellulase activity was higher after 4 days, while that of UV-C treated bananas did not differ significantly till 8 days. Interestingly, from the eighth day till the end of the experiment (18th day), the cellulase activity of CK and UV-C at the same sampling time was significantly different; the activity was significantly higher in CK as compared to UV-C group (Figure 2C). These observations suggest that after 8 days of UV-C treatment, the cellulase activity is reduced as compared to CK and it possibly maintains the hardness and the quality of the banana peels.

It is known that the total phenol content of the banana increases with maturity. In our experiment, we observed that the total phenol contents of the UV-C treated peels were higher than CK. The total phenol contents showed an upward accumulation content in the UV-C treated banana as well as CK. Overall, we observed that the phenolics contents didn’t differ significantly between UV-C and CK except on eighth day. These observations suggest that total phenols content increases with maturity and that the UV-C treatment has minor effect on the total phenols content and such an effect can be seen from day 8 (Figure 2D). On the contrary, a sharp increase in the total flavonoids content was observed with the increase in storage time (Figure 2E). UV-C treatment significantly affected (increased) the total flavonoids content in *M. nana* peels except for day 12 of storage.

#### Effect of UV-C Treatment on the Activity of Defense and Anti-Oxidant Related Enzymes

Phenylalanine ammonia lyase (PAL) plays important roles in resistance to stresses (low temperature, drought, UV irradiation) in plants. The activity of PAL increased with the prolongation of the storage time. Considering the effect of UV-C treatment, we noticed that PAL activity of the treatment group was significantly higher than control group at all time points particularly at day 8 (Figure 2F). It could be stated that UV-C treatment causes an increase in PAL activity and therefore protects banana from abiotic stresses. On the other hand, we also studied the effect of UV-C on the activity of superoxide dismutase (SOD), and peroxidase (POD), which are indispensable enzymes for anti-oxidation and anti-aging systems. The SOD activity of the two groups of banana fruits decreased briefly in the early stage of storage, and then increased significantly. During the storage period, the SOD activity of the treatment group was always higher than that of the control group, and the difference was extremely significant (*p* < 0.01). The difference was the largest on the eighth day. The SOD activity of the treatment group was 4.75 times that of the control group. (Figure 2G). The results show that UV-C treatment can effectively induce the increase of SOD activity of banana fruits, which is beneficial to the elimination of oxygen free radicals generated by the ripening and senescence during storage. The activity of the key enzyme in the antioxidant system of plants i.e., POD was increased with UV-C treatment however, the differences were not significant until day 8; significant differences were observed from day 12 and later (Figure 2H). These observations suggest that the UV-C treatment enhances the activity of POD and hence activates the antioxidant system in banana peels.

#### Effect of UV-C Treatment on ABTS, DPPH, and FRAP of *M. nana* Peels

In this experiment, three indicators i.e., 2, 20 -azinobis-(3-ethylbenzothiazoline-6-sulfonic acid (ABTS) value, DPPH, and FRAP were used to evaluate the antioxidant capacity of banana peels before and after UV-C treatment. The ABTS clearance rate of the two groups of bananas continued to increase with the days of storage. The ABTS clearance rate of the UV-C treated peels was significantly higher than that of the CK at 4, 8, and 18 days (Figure 2I). The DPPH clearance rate of the two groups of bananas continued to increase during the storage period, though on the day 0, the differences between the treated and CK group were non-significant. These observations suggest that DPPH as like ABTS clearance rate is affected by storage time as well as the UV-C treatment (Figure 2J). The FRAP values only differed significantly between UV-C and CK groups after 8 and 18 days, however, the FRAP value of the two groups of bananas increased continuously during the storage period, and that the FRAP value of the treatment group was always higher than that of the control group (Figure 2K). These observations indicate a positive impact of UV-C treatment on the antioxidant capacity of the tested bananas.

### Transcriptome Comparison of CK and UV-C

#### Transcriptome Sequencing

Based on the effect of UV-C treatment on banana peel appearance, quality, and other physiological parameters, it was concluded that storage under the experimental conditions for 8 days were suitable for maintenance of banana quality and hardness. Therefore, we further processed the banana peels treated with UV-C for 8 days (Figures 1, 2).

**FIGURE 3:**
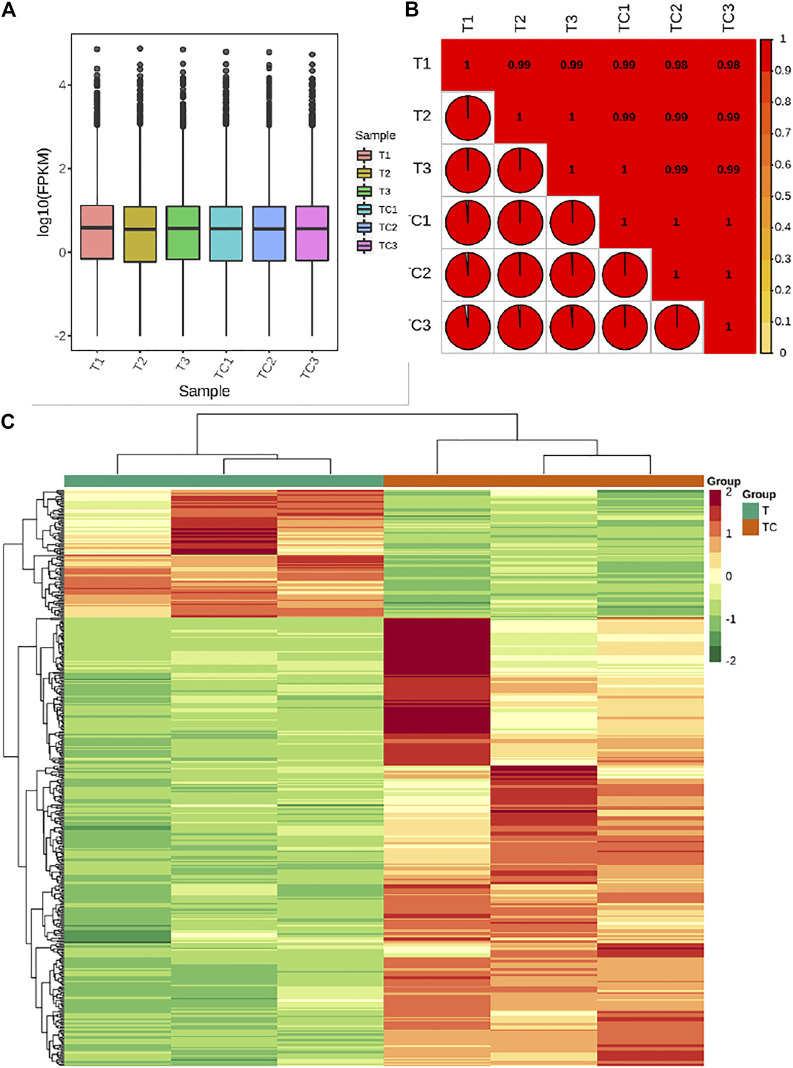
**(A)** Overall Fragments Per kilobase of Transcript per Million fragments mapped for UV-C treated *M. nana* (T) and control (CK). **(B)** Pearson Correlation Coefficient between the UV-C and CK replicates. **(C)** Heatmap and hierarchical clustering of the expression of differentially expressed genes (DEGs) between UV-C and CK bananas. Differential gene expression between UV-C and CK.

The transcriptome sequencing of six banana peel samples resulted in 48.14 Gb clean data with Q30 base % >90%. The clean reads ranged from 50,539,726 to 58,994,130 reads per sample (average 53,803,030 reads) and the average GC content per sample was 51.61% (Supplementary Table S1). Quantification of gene expression results for the UV-C treated and CK samples is shown in Figure 3A; the Fragments Per Kilobase of Transcript per Million fragments mapped (FPKM) of UV-C treated banana peels was slightly higher than CK (Figure 3A). The gene expression quantification results were highly reliable evident from higher Pearson Correlation Coefficient (PCC) between the expression of treatments and replicates (Figure 3B).

#### Differential Gene Expression Between UV-C and CK

The screening conditions for selection of the differentially expressed genes (DEGs) was log2 Fold Change ≥1 and false discovery rate (FDR) < 0.05. The screening resulted in the identification of 425 DEGs; 94 and 331 genes were downregulated and upregulated, respectively, in UV-C treated banana as compared to CK. Top-5 highly downregulated genes in UV-C treated banana were Protein of unknown function (DUF1191; *Ma11_g18320*), MYB44-like transcription factor (TF) *Ma07_g11110*, ethylene-responsive transcription factor ERF057 (*Ma04_g24880*), auxin efflux carrier component 3a-like (*Ma07_g18160*), and LOB domain-containing protein 18 (*Ma04_g39080*) (Supplementary Table S2). The changes in the expression of these genes indicate that UV-C treatment affects ethylene responses, auxin transport, responses to abiotic stresses (Jaradat et al., 2013), and possibly regulates cell wall loosening (Lee and Kim, 2013). Top-5 genes that showed higher log two fold change values (higher expression in UV-C treated banana) were glutathione S-transferase (*Ma05_g07440*), isoleucyl-tRNA synthetase (*Ma09_g10880*), Wound-induced protein (*Ma11_g17160*), alcohol-forming fatty acyl-CoA reductase (*Ma09_g30440*), and histone H3 (*Ma05_g00960*) (Supplementary Table S2). These increased expressions indicate that UV-C treated banana has a stronger wounding response (Yen et al., 2001), increased detoxification of toxic substances by their conjugation with glutathione (Yen et al., 2001), higher protein synthesis (Saga et al., 2020), higher fatty alcohol synthesis (Metz et al., 2000), and cell cycle and development (Otero et al., 2014).

KEGG pathway enrichment analysis indicated that the DEGs were significantly enriched in taurine and hypotaurine metabolism, cutin, suberine and wax biosynthesis, aminoacyl-tRNA biosynthesis, phenylpropanoid biosynthesis, flavonoid biosynthesis, and stilbenoid, diarylheptanoid and gingerol biosynthesis pathways. We also found that the DEGs were enriched in Flavone and flavonol biosynthesis, starch and sucrose metabolism, plant-hormone signal transduction, MAPK signaling pathway-plants, plant-pathogen interaction, and carotenoid biosynthesis. Detailed changes in each pathway are presented below (Supplementary Figure S1).

a. Differential regulation of signaling and defense related pathways

Our results showed that the DEGs were enriched in two signaling related pathways i.e., plant-hormone signaling (17 DEGs) and MAPK signaling pathway (13 DEGs). Four DEGs i.e., auxin response factor (ARF), two jasmonate ZIM domain-containing protein (JAZ), and a protein brassinosteroid insensitive 1 (*BRI1*) had lower expression in UV-C treated bananas. The expression of three JAZs; two downregulated and one upregulated in UV-C treated bananas, indicate that Jasmonic acid (JA) signaling is affected by UV-C irradiation. Other genes in JA signaling i.e., coronatine-insensitive protein 1 (*COI1*), JA-amino synthetase (*JAR1*), and two *MYC2* genes were also upregulated in UV-C treated banana peels. The UV-C irradiation on banana resulted in the increased expression of gibberellin (gibberellin receptor *GID1* and DELLA protein), abscisic acid (ABA, PYR/PYL family protein and protein phosphatase 2C, *PP2C*), ethylene (ethylene insensitive protein 3, *EIN3* and ethylene-responsive transcription factor 1, *ERF1*), and salicylic acid (SA, pathogenesis-related protein 1, *PR1*) signaling related genes. Therefore, it could be suggested that the UV-C irradiation activates the phytohormone signaling mechanisms in banana peels. These changes affect fruit ripening and senescence possibly due to changes in the expression of *EIN3* and *ERF1* (Lado et al., 2015). Additionally, the UV-C treatment impart changes in anthocyanin accumulation in fruits in response to differential expression of PYR/PYL family protein (Gao et al., 2018; Yang et al., 2019). The delay in softening is possibly due to changes in the expression of gibberellin signaling related genes (Wongs-Aree et al., 2014). Finally, UV-C treatment may also help in maintaining fruit quality by activating SA related genes (Ennab et al., 2020) (Supplementary Table S3).

The *MYC2* TFs, *EIN3*, *ERF1*, *PP2C*, *PYR/PYL*, and *PR1* genes were also significantly enriched in the MAPK signaling. Among other genes that showed increased expression in response to UV-C irradiation, we found two calmodulin (*CaM4*, *Ma00_g02460* and *Ma00_g06660*), a *WRKY22* (*Ma04_g08390*), respiratory burst oxidase (*RbohD*, *Ma05_g29360*), LRR receptor-like serine/threonine-protein kinase ERECTA (*Ma10_g00450*), and transmembrane protein 222 (*Ma04_g08810*). Increased expression of *WRKY22* suggests that reactive oxygen species (ROS) are induced in response to UV-C treatment in banana peel as compared to CK (Adachi et al., 2015). While the increased expressions of CaM4s and RbohD maintains the homeostasis of ROS in UV-C treated peels (Taj et al., 2010; Pathak et al., 2013) (Supplementary Table S3). Thus, UV-C treatment protects bananas from decay.

Nineteen DEGs were enriched in plant-pathogen interaction pathway; only two DEGs (3-ketoacyl-CoA synthases, *Ma04_g08880* and *Ma03_g01910*) showed reduced expression after UV-C treatment as compared to control. However, two other genes annotated as 3-ketoacyl-CoA synthase (*Ma02_g23450* and *Ma03_g02070*) were upregulated. Genes that were enriched in the MAPK signaling pathway were also enriched in the plant-pathogen interaction pathway i.e., *RbohD*, *WRKY22*, *CaM4*, and *PR1*. Most importantly, we noticed the UV-C treatment increased the expression of pattern recognition receptors (PRRs i.e., *EIX44198* and *CERK1* (chitin elicitor receptor kinase 1)), genes related to hypersensitive response (PBS1, serine/threonine-protein kinase *PBS1*, and *HSP90*, molecular chaperone *HtpG*), and pathogenesis-related genes transcriptional activator *PTI5* (Supplementary Table S3). These expression changes suggest that UV-C treatment induced a hypersensitive response in banana peels as compared to CK and thus protects banana during storage.

Other enzymes that are parts of antioxidant and defense responses i.e., PAL (*Ma01_g04420* and *Ma05_g03720*) and POD (*Ma10_g05180, Ma07_g23680,* and *Ma11_g04630*) also showed increased expressions after UV-C treatment. These observations also confirm the activity of PAL and POD as presented in Figure 2. Thus, UV-C treatment activates a defense response in banana peels.

b. Effect of UV-C treatment on secondary metabolites related pathways

**FIGURE 4:**
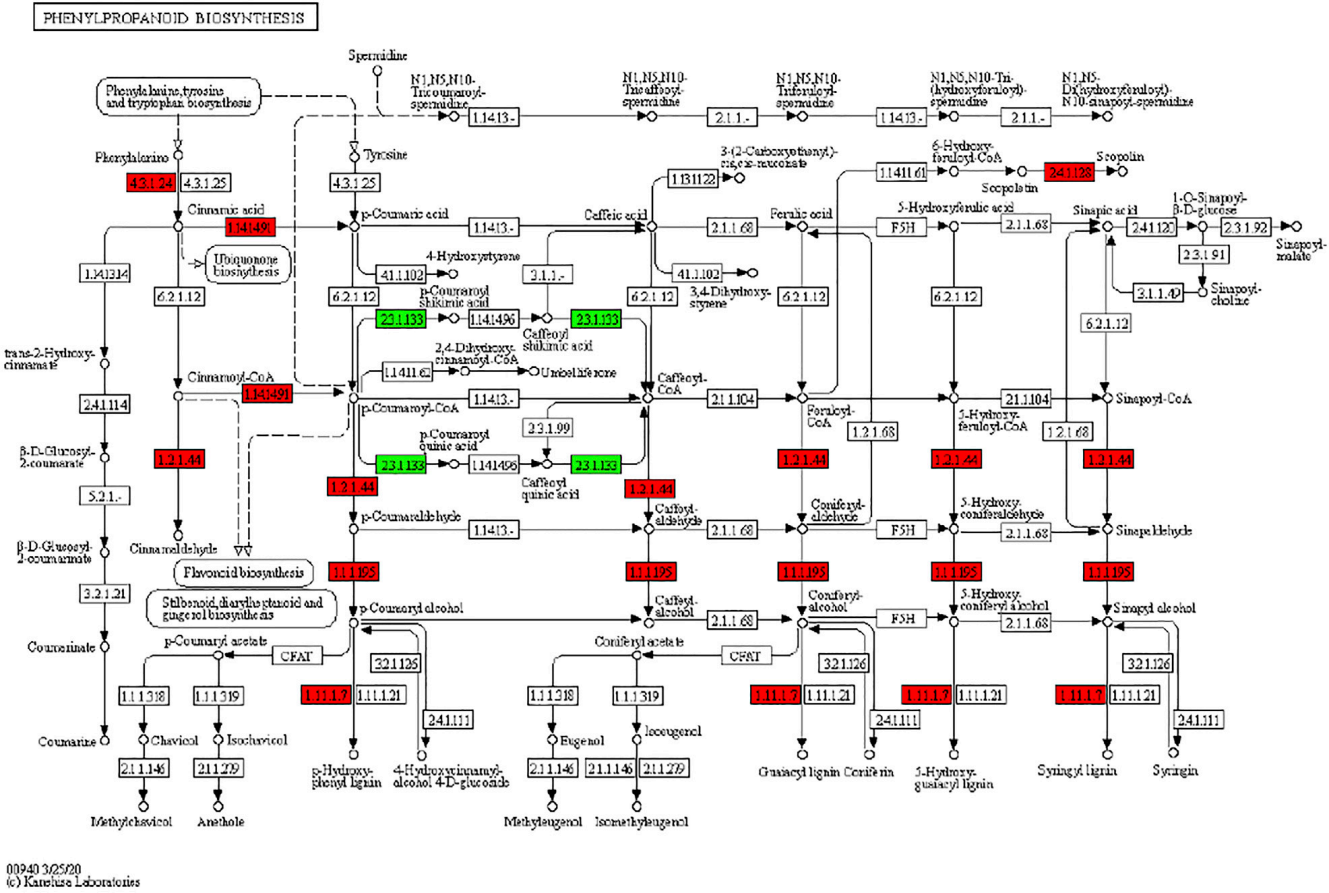
Differential regulation of phenylpropanoid biosynthesis pathway in *M. nana* peel under the influence of UV-C irradiation as compared to control. Number in the boxes represent E.C. numbers of the enzymes. Red and green color indicates upregulation and downregulation of genes in response to UV-C treatment.

**FIGURE 5:**
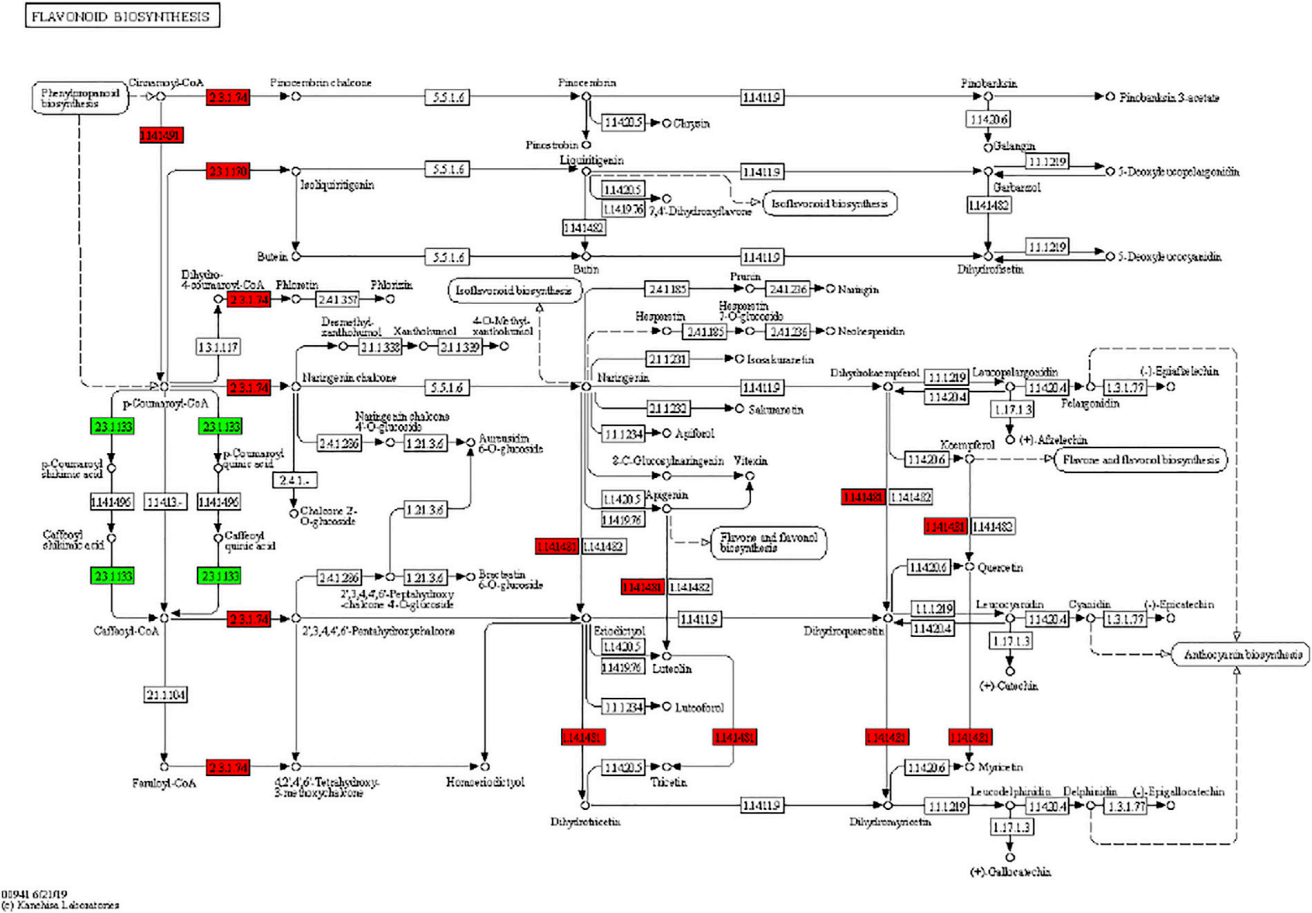
Differential regulation of flavonoid biosynthesis pathway in banana peels treated with or without UV-C irradiation. Number in the boxes represent E.C. numbers of the enzymes. Red and green color indicates upregulation and downregulation of genes in response to UV-C treatment.

It has been reported that secondary metabolite biosynthesis increases when tomato is treated with UV-C irradiation (Liu et al., 2018). To this regard, our findings that DEGs were enriched in many secondary metabolite related pathways is quite relevant. Seven DEGs annotated as cysteamine dioxygenases (enriched in taurine and hypertaurine biosynthesis metabolism) showed increased expression in UV-C treated banana peels as compared to CK. These genes are known for their role in enhanced plant survival under abiotic stresses (Licausi et al., 2011) by increasing the biosynthesis of taurine (Kotzamanis et al., 2020). Hence, it is possible that UV-C treatment affected their expression in order to respond to changes in O_2_ levels in peels, since cysteamine dioxygenases have also been shown to be sensitive to O_2_ levels (White et al., 2018). Their function is directly linked with ERFs (White et al., 2020), thus it could be suggested that together, ERFs and cysteamine dioxygenases are activated under the influence of UV-C in banana peel and protect it during storage. Among other pathways, we found that 13 DEGs were enriched in the phenylpropanoid biosynthesis pathway. Only one gene i.e., shikimate O-hydroxycinnamoyltransferase (*Ma03_g26620*) was downregulated in response to UV-C treatment, while all other genes showed higher expression in the UV-C group; three PODs, three trans-cinnamate 4-monooxygenases, two cinnamoy-CoA reductases, and two PALs (Figure 4). The higher expression of all these genes leads towards increased biosynthesis of lignins (p-hydroxyphenyl lignin, guaiacyl lignin, coniferin, and syringyl lignin). The activity of callose (Figure 2C) is also in line with these observations. The three trans-cinnamate 4-monooxygenases were also enriched in flavonoid biosynthesis pathway and stilbenoid, diarylheptanoid and gingerol biosynthesis (Figure 5; Supplementary Table S3). Furthermore, the UV-C treatment resulted in increased expression of two chalcone synthases, a shikimate O-hydroxycinnamoyltransferase (also enriched in stilbenoid, diarylheptanoid and gingerol biosynthesis), and a flavonoid 3′,5′-hydroxylase. All these genes are located in the upstream of anthocyanin biosynthesis, dihydrotricetin and dihydromyricetin biosynthesis. These expression changes suggest that UV-C treatment increases the production of these compounds which are substrates for production of unstable leucopelargonidin, leucocyanidin, and leucodelphinidin. However, we did not detect the differential expression of any of the genes in anthocyanin biosynthesis pathway (Hua et al., 2013). Thus, specifically, overproduction of dihydrotricetin and dihydromyricetin in banana peels under UV-C treatment might be for different protective activities. However, we detected the increased expression of zeaxanthin epoxidase (*Ma07_g03980*) and (+)-abscisic acid 8′-hydroxylase (*Ma07_g07210*). Zeaxanthin epoxidase converts zeaxanthin into antheraxanthin and then to violaxanthin and protects plants against damage by excess light (Hieber et al., 2000).

c. Effect of UV-C treatment on other important pathways

The KEGG enrichment analysis also revealed that the DEGs were significantly enriched in cutin, suberine and wax biosynthesis and aminoacyl-tRNA biosynthesis. Seven DEGs were enriched in cutin, suberine and wax biosynthesis. A gene annotated as fatty acid omega-hydroxylase (*Ma03_g26150*) was upregulated in UV-C treated peels. It converts 9,10-epoxy-18-hydroxystearate into 9,10,18-Trihydroxystrearate (an unsaturated fatty acid, UFA). Previously, it was shown that the proportion of UFAs in peels is closely related with membrane fluidity and tolerance against abiotic stresses. A higher proportion of UFAs is found in plants that show tolerance to cold (Liang et al., 2020). Thus, it could be proposed that UV-C irradiation increases the UFA content in banana peel. Further, we found four DEGs (three alcohol-forming fatty acyl-CoA reductases and an aldehyde decarbonylase) that were upregulated in response to UV-C irradiation. Both genes control essential steps in the biosynthesis of wax (Cheesbrough and Kolattukudy, 1984; Metz et al., 2000). Higher wax production in fruit offers a better shelf-life; cuticle is known as a modulator of postharvest quality of fruits (Lara et al., 2014; Lara et al., 2019).

Apart from wax and UFA biosynthesis, we found that 10 DEGs were enriched in aminoacyl-tRNA biosynthesis pathway (Supplementary Table S3). Interestingly, all the genes were annotated as isoleucyl-tRNA synthetases. These genes are known to play essential roles in plants e.g., (together with dirigent proteins) maintenance of plant cell wall integrity (Paniagua et al., 2017). Thus, it is possible that UV-C treatment increases the expression of isoleucyl-tRNA synthetases, which in turn helps banana to maintain cell wall to avoid being flaccid.

#### qRT-PCR Analysis of Selected DEGs

**FIGURE 6:**
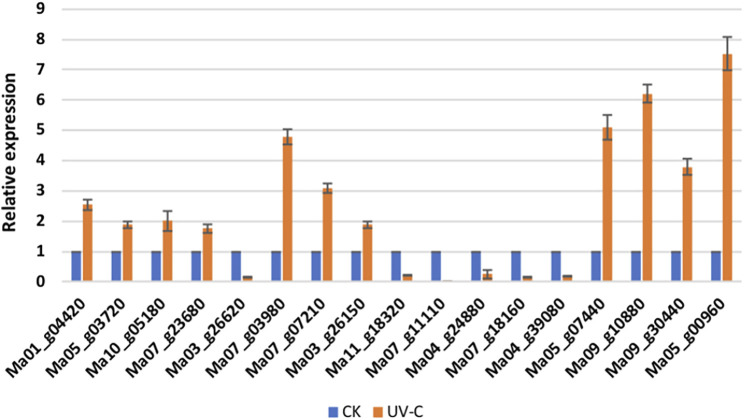
qRT-PCR analysis of selected *M. nana* genes that were differentially expressed in UV-C treated peels (UV-C) as compared to control (CK). The error bars on columns represent the standard deviation.

The qRT-PCR analysis of 17 *M. nana* genes showed that the expression profiles were consistent with the transcriptome sequencing results in *M. nana* peels treated with UV-C as compared to CK (Figure 6). These results validated the RNA sequencing data and confirmed its reliability.

#### Metabolome Analysis of UV-C Treated *M. nana* vs CK

The metabolome analysis resulted in the identification of 38 differentially accumulated metabolites (DAMs) (Table 1). The DAMs belonged to a range of metabolite classes i.e., alkaloids, amino acids and derivatives, anthocyanins, diterpenoids, flavanols, flavonoids, flavonols, free fatty acids, glycerol ester, phenolic acids, tannin, and xanthone. Eight of the 38 metabolites were accumulated in lower quantities in UV-C treated bananas as compared to CK. These DAMs included two amino acids i.e., L-tryptophan and L-valyl-L-Phenylalanine, three free fatty acids, a glycerol ester, a phenolic acid (disooctyl phthalate), and a tannin (cinnamtannin A2). Interestingly, all alkaloids, flavonoids, and terpenoids showed increased accumulation in banana peels treated with UV-C irradiation (Table 1). These observations are in accordance with the results of total phenols and flavonoids contents (Figures 2D,E). Top five DAMs that were accumulated in response to UV-C treated are 4-p-Cumaroyl-rhamnosyl-(1→6)-D-glucose, 5-Aminovaleric acid, 6,8-dihydroxy-1,2,3-trimethoxyxanthone, quercetin-3-O-(2'''-p-coumaroyl) sophoroside-7-O-glucoside, and Hydroxyanigorufone (Table 1).

**TABLE 1:** List of metabolites that were differentially accumulated in *Musa nana* peel before and after UV-C treatment.

Compounds	Index	CK (ion intensity)	UV-C (ion intensity)	VIP (variable importance in projection)	Log2FC
Alkaloids					
3-Carbamyl-1-methylpyridinium (1-Methylnicotinamide)	pme1738	18,232	37,087	1.04	1.02
Amino acids and derivatives					
5-Aminovaleric acid	pme0120	9	24,860	1.67	11.43
L-Tryptophan	mws0282	2,257,100	1,128,307	1.58	-1.00
L-Valyl-L-Phenylalanine	Lmhp002001	29,604	11,240	1.47	-1.40
Anthocyanins					
Cyanidin-3-O-(6″-O-*p*-Coumaroyl) glucoside	Lmpp003789	8,470,067	19,123,333	1.63	1.17
Delphinidin-3-O-(6″-O-*p*-coumaroyl)glucoside	Lmpp003662	12,015,000	27,235,000	1.62	1.18
Diterpenoids					
Cafestol	pme3459	25,221	61,500	1.62	1.29
Flavonols					
7-O-Galloyltricetiflavan	Lmhn004960	20,380	57,734	1.33	1.50
Catechin gallate	mws0355	28,317	78,391	1.17	1.47
Epigallocatechin-3-gallate	mws0034	62,222	1,72,137	1.25	1.47
Flavonoid					
Luteolin-7-O-neohesperidoside (Lonicerin)	pmp001079	26,332	64,707	1.65	1.30
Hesperetin-8-C-glucoside-3′-O-glucoside*	pmb0618	62,916	1,38,263	1.60	1.14
Flavonols					
6-Hydroxykaempferol-7-O-glucoside	pmp001309	3,419,267	6,891,400	1.30	1.01
Quercetin-3-O-(6″-acetyl)galactoside	Hmln002199	1979	7,946	1.48	2.01
Quercetin-7-O-(6″-malonyl)glucoside	pmp000589	15,604	36,131	1.56	1.21
Kaempferol-3-O-glucoside-7-O-rhamnoside	Lmsp004670	7,849,067	19,585,000	1.58	1.32
Kaempferol-3-O-neohesperidoside*	Lmjp002867	7,249,867	18,705,333	1.61	1.37
Quercetin-3-O-apiosyl (1→2)galactoside	Lmtp004044	3,513	15,564	1.01	2.15
Quercetin-3-O-glucoside-7-O-rhamnoside	Lmsp004166	12,898,333	26,437,667	1.62	1.04
Quercetin-7-O-rutinoside	pmb0711	10,995,400	24,707,000	1.55	1.17
Quercetin-3-O-rutinoside (Rutin)	mws0059	4,823,300	1,1,302,000	1.55	1.23
6-Hydroxykaempferol-3,6-O-Diglucoside	pmp001310	32,312	72,397	1.56	1.16
Kaempferol-3-O-neohesperidoside-7-O-glucoside	pmp001105	1,49,923	3,41,013	1.63	1.19
Quercetin-3-O-rutinoside-7-O-glucoside	Lmmp002334	1,90,217	4,25,890	1.59	1.16
Quercetin-3-O-(2‴-*p*-coumaroyl)sophoroside-7-O-glucoside	Lmwp004293	8,003	62,906	1.62	2.97
Quercetin-3-O-(6″-feruloyl)glucoside-7-O-rutinoside	Lmdp004461	5,339	12,901	1.62	1.27
Free fatty acids					
Palmitoleic Acid	mws0361	6,847	2,752	1.27	-1.32
9-Hydroperoxy-10E,12,15Z-octadecatrienoic acid	pmb2791	1,26,204	15,634	1.33	-3.01
13S-Hydroperoxy-6Z,9Z,11E-octadecatrienoic acid	pmb2789	1,80,035	24,968	1.30	-2.85
Glycerol ester					
PI(18:2/0:0)	Lmqn008369	53,432	25,019	1.65	-1.09
Others					
Hydroxyanigorufone	HJAP129	3,28,233	2,456,067	1.59	2.90
3,5,7,4′-Tetrahydroxy-Coumaronochromone	Hmyp002315	61,487	1,90,140	1.64	1.63
Phenolic acids					
Diisooctyl Phthalate	Lmmp010562	4,090,667	8,67,850	1.22	-2.24
4-*p*-Cumaroyl-rhamnosyl-(1→6)-D-glucose	Zmhn002508	9	32,638	1.67	11.82
Di-O-Glucosylquinic acid	Zmhn000785	46,582	1,14,807	1.66	1.30
Syringic acid-4-O-(6″-feruloyl) glucoside	pmb0824	6,162	12,872	1.60	1.06
Tannin					
Cinnamtannin A2	pmn001649	11,418	5,410	1.55	-1.08
Xanthone					
6,8-Dihydroxy-1,2,3-trimethoxyxanthone	pmp001011	2,687	34,100	1.54	3.67

**FIGURE 7:**
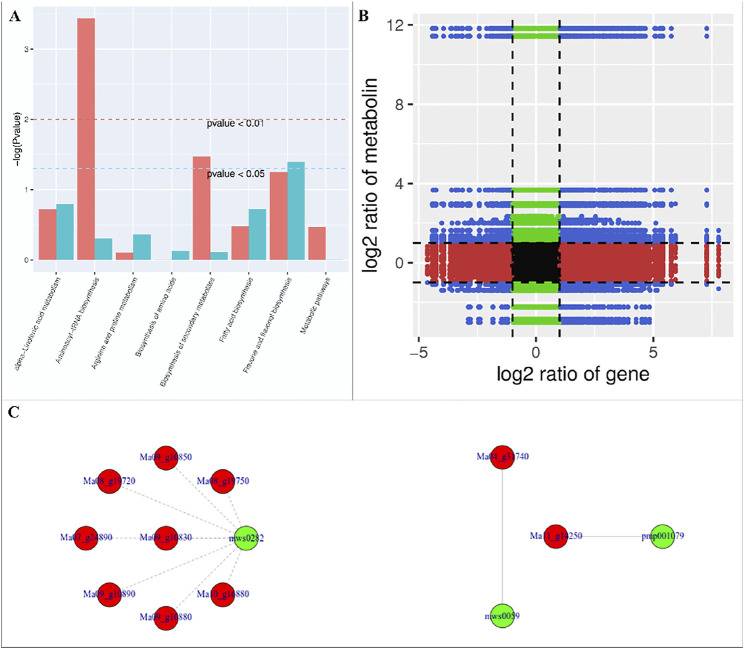
**(A)** Co-joint analyses of DEGs and DAMs between UV-C treated M. nana and CK. **(A)** KEGG pathway enrichement, **(B)** nine-quadrant graph, and **(C)** network diagrams of DAMs and DEGs based on PCC (≥0.8).

We found that the DAMs were significantly enriched in eighteen different pathways (Supplementary Figure S2). Significant enrichment of DAMs was observed in anthocyanin biosynthesis (cyanidin-3-O-(6″-O-p-Coumaroyl) glucoside and delphinidin-3-O-(6″-O-p-coumaroyl) glucoside) and flavone and flavonol biosynthesis (quercetin-3-O-rutinoside (Rutin) and quercetin-3-O-(6″-feruloyl) glucoside-7-O-rutinoside) pathways. The changes in the accumulation pattern of the anthocyanin biosynthesis pathway related metabolites are consistent with the increased expression of the genes that were present upstream in this pathway (Figure 5). Thus, the changes in the expression of phenylpropanoid pathway genes and accumulation of these metabolites might be a reason for the color differences between the UV-C and CK bananas. Similarly, the higher accumulation of the two metabolites in flavone and flavonol biosynthesis pathway is consistent with the observed higher flavonoid contents in UV-C treated bananas (Figure 2E). The enrichment of DAMs in other pathways is consistent with that of DEGs’ enrichment in KEGG pathways (Supplementary Figures S1-2; Figure 7A).

### Co-Joint Analysis of DEGs and DAMs

The nine-quadrant graph based on the PCC between DEGs and DAMs (PCC ≥0.8) was generated. Within the nine-quadrant graph, we looked at the third and seventh quadrant since the genes and metabolites in these quadrant have consistent regulatory trend in response to UV-C treatment (Figure 7B). The DEGs and DAMs in these two quadrant have positive correlation and hence it is possible that the DAMs are regulated by the DEGs within these quadrants. These genes and metabolites are presented in Supplementary Table S4. The newtwork analysis based on PCC indicated that a metabolite mws0282 (L-Tryptophan) had negative correlation with isoleucyl-tRNA synthetases (Figure 7C first panel). Similarly, luteolin-7-O-neohesperidoside (Lonicerin) had positive correlation (0.92) with flavonoid 3′,5′-hydroxylase (Ma11_g14250) and quercetin-3-O-rutinoside (Rutin) had positive correlation (0.974) with isoflavone 7-O-glucoside-6″-O-malonyltransferase (Ma04_g31740) (Figure 7C). These combined analyses indicate that UV-C irradiation had impacted the flavonoid as well as phenylpropanoid biosynthesis pathways, which is supported by the individual omics analyses.

## Discussion

### UV-C Treatment Affects *M. nana* Fruit Quality and Antioxidant Capacity

Increasing attention is being paid to the effects of UV irradiation on the postharvest preservation of fruits and vegetables. Studies have shown that the UV light improves the quality and shelf life of fruits e.g., strawberry, grapes, mango, and pineapple [ (Zhang and Jiang, 2019) and references therein]. Our observations that the decay rate and weight loss rate was significantly lower in UV-C treated bananas is consistent with earlier reports on tomato and strawberries (Cote et al., 2013). This is possibly due the higher DPPH and ABTS free radical scavenging ability as well as increased FRAP value (Figure 2). Earlier it was reported that antioxidant properties and postharvest life of exotic Andean fruits were increased when treated with UV-C irradiation (Andrade-Cuvi et al., 2017). This is not only the case of banana and other fruits, but a study showed that the bioactive components and antioxidant properties of *Clerodendrum volubile* leaves was increased when treated with UV-C irradiation (Adetuyi et al., 2020). Another possibility of reduced decay rate of UV-C treated banana might be the lower activity of cellulase (Figure 2C). Cellulases are known for their ability to decompose cellulose and other polysaccharides and a positive correlation has been observed between cellulase activity and fruit firmness in muskmelons (Elhassan and Abu-Goukh, 2016). These results are also consistent with the increased expression of genes enriched in the phenylpropanoid biosynthesis pathway. Possibly, UV-C treatment induce the phenylpropanoid biosynthesis pathway in order to keep the peel healthy and rigid by increased biosynthesis of lignin. We say this because it has been reported that induced transcriptional profiling of phenylpropanoid pathway genes resulted in increased lignin content (Ali and McNear, 2014). Also, it is has long been known that UV treatment induces phenylpropanoid biosynthetic enzymes (Walter, 1989). Thus, increased antioxidant potential and higher cellulase activity (together with increased expression of phenylpropanoid biosynthesis pathway genes) could be related to the improved fruit quality (lower fruit decay and weight loss) in UV-C treated banana.

### UV-C Treatment Increases the Flavonoid and Phenolic Content in *M. nana*

The application of low doses of UV-C irradiation on vegetables and fruits can induce specific secondary metabolite biosynthesis (Cisneros Zevallos, 2003). For example, earlier reports suggested that UV treatment triggered the accumulation of phytochemicals e.g., total phenolics, carotenoids, and phytoalexins (Alothman et al., 2009b; Olaiya et al., 2016). Our results that the UV-C treatment resulted in increased total phenolics and total flavonoid contents are consistent with these earlier reports (Figures 2D,E). This was further confirmed by the results of multi-omics analyses (Supplementary Table S3; Table 1). Since flavonoids (especially flavonols) are affective scavengers of ROS (Falcone Ferreyra et al., 2012), it is not surprising that the biosynthesis and accumulation of these compounds was strongly induced in UV-C treated bananas. This is true for all UV lights especially for UV-B and UV-C. However, UV-C is more energetic and most of the postharvest UV treatments are carried out with UV-C (Walter, 1989; Allende and Artés, 2003; Alothman et al., 2009b). Increased expression of the genes in both phenylpropanoid biosynthesis and flavonoid biosynthesis pathways and concomitant accumulation of metabolites is consistent with the summarized reviewed by Zoratti*, et al.* (Zoratti et al., 2014). The activity of the genes in these pathways have also been reported to be regulated by MYB TFs i.e., MYBs may activate or repress their activities. A similar mechanism might be active in UV-C treated banana since we found the differential expression of 10 MYB TFs in UV-C vs CK bananas (Supplementary Table S2). The MYB TFs that regulate flavonoid biosynthesis have been reported to interact with bHLH and WD40 -repeat proteins to form a complex known as MBW-regulatory complex [54 and references therein]. Our transcriptome expression analyses showed the increased expression of three bHLH TFs (*Ma07_g02820*, *Ma03_g17620*, and *Ma08_g34470*) in UV-C treated bananas (Supplementary Table S2). However, we did not find differential expression of any WD40. A similar observation was reported in nectarin (*Prunus persica*), where authors reported the upregulation of bHLH and not the WD40 in light treated fruits (Ravaglia et al., 2013). Thus, it is possibly that in UV-C treated bananas the increased expression and accumulation of flavonoids could be regulated only by the MYBs and bHLH TFs. MYB TFs specifically interact with a MYB recognition element present in the promoter region of flavonoid biosynthesis genes e.g., chalcone synthases (Feldbrügge et al., 1997). UV-C treatment also increased the expression of two chalcone synthases (*Ma05_g32080* and *Ma03_g01020*) (Supplementary Table S2) supporting our proposal that UV-C treatment regulates the expression and accumulation of flavonoids in banana peel by MYB TFs. Specific temporal and spatial expression characterization of these genes in response to UV-C would be a promising future study.

### UV-C Treatment Activates Phytohormone Signaling Pathways in *M. nana*

Decades of research has shown that hormones have significant roles in fruit ripening and the endogenous/exogenous phytohormones continue to play roles in controlling the physiological events in postharvest stored fruits (Ludford, 1995). The postharvest application of UV-C irradiation on fruits is a known factor in slowing down the ripening and senescence process as well as decay (Rivera-Pastrana et al., 2007). These changes are directly associated with the signaling mechanisms such as plant-hormone signaling as well as MAPK signaling since both pathways play roles in wounding responses, defense responses, growth, cell division, fruit ripening, and senescence and related mechanisms (Kumar et al., 2019; Wang et al., 2020b; Gouda et al., 2020). Our observations that the expression of ARF and BRI1 indicates that cell enlargement, cell elongation, and plant growth was reduced. It is also known that BRIs play roles in fruit quality and post-harvest storage, therefore, a similar role of BRI1 could be expected in UV-C treated banana (Roghabadi and Pakkish, 2014; Champa et al., 2015). UV-C treatment also initiated JA signaling in banana as evidenced from the differential expression of JAZs and the increased expression of COI1, JAR1, and MYC2 genes. The activation of these genes affects indole alkaloid biosynthesis, monoterpene biosynthesis, and ubiquitin mediated proteolysis (Katsir et al., 2008). These results are consistent with the accumulation of 3-Carbamyl-1-methylpyridinium(1-Methylnicotinamide) (an alkaloid) in UV-C treated banana. Therefore, UV-C irradiation activates JA signaling by increasing the expression genes that control several steps in the pathway and resultantly increases alkaloid accumulation (Goldhaber-Pasillas et al., 2014; Xu et al., 2015). Apart from JA signaling, another possible reasons of the observed physiological and visual changes could be the increased expression of GID1 and DELLA in UV-C treated bananas (Supplementary Table S3). Previous reports suggested that UV-B inhibits the leaf growth in maize (*Zea mays*) by modulating the gibberellin levels (Fina et al., 2017). Similar roles might be possible in UV-C treated bananas. Specific roles of gibberellins in UV-C treated postharvest bananas would be an interesting topic. It is important to state that the delay in softening of banana peels in response to UV-C treatment could be due to the changes in the expression of the gibberellin signaling related genes (Wongs-Aree et al., 2014). The delayed ripening and reduced decay rate could also be due to UV-C induced ABA signaling as we found the higher expression of PYR/PYL and PP2C proteins in UV-C group (Supplementary Table S3). This proposition is consistent with the observations in strawberry, where the authors reported that UV-C treatment affects the ABA-induced physiological changes (Li et al., 2014). Finally, our results that the UV-C irradiation activated EIN3 and ERF1 are in accordance with Tiecher*, et al.* (Tiecher et al., 2013). They reported that postharvest UV-C treatment induced ethylene production in tomato. The EIN3 gene targets ERF1, which is further involved in a range of ethylene responses (Solano et al., 1998). Other studies have also reported that UV irradiation upregulates the ethylene related genes e.g., 1-aminocyclopropane-1-carboxylic acid synthases (An et al., 2006). Also, it is known that EIN3 also directly regulates the SA biosynthesis (Chen et al., 2009), which plays role in defense responses (see next section) (Tian et al., 2014). At least, it is known that the activation of SA related genes helps in maintain fruit quality, thus the increased expression of PR1 suggests that UV-C treatment helps banana to maintain quality during storage (Tian et al., 2014). Taken together, our results revealed that UV-C treatment increases the expression of phytohormone signaling related pathways.

### UV-C Treatment Differentially Regulates Defense Responses in *M. nana*

Bananas have shorter shelf-life and therefore their degradation occur during storage due to the accumulation of ROS and enzymatic oxidation of polyphenols. These changes result in color changes, accumulation of certain compounds i.e., superoxide anion, H_2_O_2_, thiobarbituric reaction compound, and other reactive species (Murmu and Mishra, 2018). The use of UV-C treatment in postharvest storage of different fruits is increasing (Pongprasert et al., 2011; Pinheiro et al., 2015; Xu and Liu, 2017). Our results found that UV-C treatment differentially regulated defense responses in bananas as compared to control. Mainly, we found that UV-C treatment increased the expression of different genes controlling key steps in MAPK signaling (plant) and plant-pathogen interaction pathways (Supplementary Table S3). First of all, the UV-C irradiation initiated a hypersensitive response (HR) in banana peels by increasing the expression of CaM4 and RbohD (Chen and Yang, 2019). Secondly, the UV-C treatment also increased the expression of Pti5 and WRKY22, which are known to induce the expression of defense related genes i.e., PR1. Since, we saw an increase in the expression of Pti5, WRKY22, and PR1 in response to UV-C (Supplementary Table S3), therefore, it could be stated that UV-C treatment initiates a defense response in banana peels to keep them disease free and fresh for longer time. A similar function of UV treatment (particularly for UV-B) was reported that it causes the generation of ROS, which stimulates PR1 (Green and Fluhr, 1995). The increased expression of two PRRs, and genes associated with effector-triggered immunity i.e., PBS1 and HSP90 also play role in protecting the UV-C treated banana from decay (Kishimoto et al., 2011; Balint Kurti, 2019). However, from another perspective, the UV-C irradiation itself could be a stress and the defense response might be activated since it has been reported that MAPK cascade can help plants by providing tolerance against high UV doses (Wituszyńska and Karpiński, 2013). Considering this, a cross-talk between the defense against abiotic and biotic stress during storage with or without UV-C treatment would remain an active topic.

## Materials and Methods

### Plant Material and Experimental Conditions

Eight weeks old green-ripe bananas (*Musa nana* Lour) were picked from an orchard in Yangchun City, Guangdong Province and originally provided by Guangdong Ocean University. No permissions are necessary to collect such samples. The formal identification of the sample was conducted by Prof Xiaoming Qin and no voucher specimens have been deposited. Care was taken during sampling so that disease and damage free bananas of the same size were selected and transported back to the laboratory on the same day. The stalks were removed and bananas were separated. The separated bananas were washed under tap water to remove any physical impurities present on the surface of the banana peels. The bananas were then rinsed with distilled water thrice and dried under fan. Bananas were then randomly assigned to two groups i.e., control (CK) and the experimental group (UV-C). In a preliminary experiment the experimental group was then treated with six different ultraviolet (UV) doses (radiation intensity) 0.01, 0.02, 0.03, 0.04, 0.06, 0.08 KJ/m^2^ for 0–7 days (data not shown). It was found that after storage at 25°C for 5 days treated with UV-C intensities 0.06 and 0.08 KJ/m^2^ showed obvious browning of banana skin. On the contrary, 0.01 and 0.02 KJ/m^2^ had no browning. Considering this, and previous reports (Ding and Ling, 2014) we selected 0.02 KJ/m^2^ as the best UV-C treatment for banana. Therefore, we considered treating banana with 0.02 KJ/m^2^ dose. A UV-C lamp (Philips, 20 W) was used for the irradiation. Once, the energy was stabilized, the experimental group was treated with UV-C and kept under constant temperature in a humidity box (temperature 25°C, humidity 85%) and protected from light. The control and experimental groups were then observed for their appearance and storage effects after 0, 4, 8, 12, 15, and 18 days. The samples were taken at exact sampling times, peels removed and chopped, pre-cooled in liquid nitrogen, and stored in ultra-low temperature refrigerator (-80°C) for further analyses.

### Physiological Index Analyses

#### Determination of Decay Rate and Weight Loss Rate

In this experiment, the fruit decay rate and weight loss rate were used to evaluate the storage effect, which were calculated according to formulas (1) and (2) respectively. The decay rate is calculated as rot, which is based on the visible rot spots of the stored banana fruit (Gol and Ramana Rao, 2011).

$$Decay\;rate=\frac{Number\;of\;rotten\;fruits}{Total\;number\;of\;experimental\;fruits}\times 100$$ (1)

$$Weight\;loss\;rate=\frac{Quality\;before\;storage-quality\;after\;storage}{Quality\;before\;storage}\times 100$$ (2)

#### Determination of Callose Synthase, PAL, SOD, and POD Activities

Callose synthase and PAL activity was measured as reported earlier by Cao*, et al.* (Cao et al., 2007). Similarly, the activities of SOD and POD enzymes were assayed according to modified method of Oracz*, et al.* (Oracz et al., 2009) and Sun*, et al.* (Sun et al., 2011).

#### Determination of Total Flavonoid, Total Phenols, DPPH and ABTS Free Radical Scavenging Ability, and FRAP Value

Methanol extract was prepared according to the modified method described by (Silva and Sirasa, 2018). 2ml 80% methanol was added to 0.50 g of banana fruit peel powder and placed in a water bath at 60°C at 50 r/min for 25 min, then centrifuge at 5,000 r/min for 10 min, collect the supernatant, and repeat the above steps twice. The supernatant of both extractions was combined, diluted to 10 ml with 80% methanol and stored at -20°C. The extract was then used to determine total phenols, total flavonoids, DPPH free radical scavenging ability, ABTS free radical scavenging ability, and ferric reducing antioxidant power (FRAP) value. Care was taken while storing and processing the extract by covering the tubers with aluminum foil to prevent the extract from being damaged by light. Total phenols content and DPPH free radical scavenging ability was measured according the method described by Khaliq*, et al.* (Khaliq et al., 2016). Total flavonoid content, ABTS free radical scavenging ability, and FRAP value were measured according to the methods described by Al Amri and Hossain (Amri and Hossain, 2018), Thaipong*, et al.* (Thaipong et al., 2006), and Wu*, et al.* (Wu et al., 2006), respectively.

### Transcriptome Analysis

#### RNA Extraction, cDNA Library Preparation, and on Machine Sequencing

Triplicate banana samples treated with UV-C for 8 days and the respective controls were used for RNA-sequencing. Total RNA extraction, determination of purity of extracted RNAs, and RNA quantification was done as reported earlier (Chen et al., 2019).

First strand cDNA was prepared from the purified RNA of each of three replicates of the control and the experimental groups. The cDNA synthesis, and library preparation was completed as reported earlier (Chen et al., 2019). The Agilent 2,100 Bioanalyzer was used to detect the size and concentration of the library fragments. Single-stranded circular DNA molecules were used to generate DNA nanospheres (DNB), which contained more than 200 copies. The DNBs were added into the mesh holes on the chip using high-density DNA nanochip technology. Sequencing was performed by the combined probe anchored polymerization technology (cPAS) to obtain a sequencing read length of 150 bp.

#### Sequencing Data Analyses

The raw reads were filtered, sequencing error rate distribution check was performed, followed by performing a GC content check. HISAT2 was then used to compare the clean reads with the reference genome (Busche et al., 2020). Gene expression was quantified as FPKM, which was then used for subsequent analyses. The overall distribution of the FPKM was visualized and used to calculate PCC between treatments and replicates. We employed DESeq2 to obtain DEGs between UV-C and CK groups. FeatureCounts was used for count of gene reads, and then Benjamini-Hochberg method was used to perform multiple hypothesis test correction on the hypothesis test probability (*p* value) to obtain the FDR. The screening conditions for differential genes were |log2 Fold Change| ≥ 1, and FDR <0.05. Visualization, cluster analysis, and heatmap representation of the gene expression was done as reported earlier (Chen et al., 2019).

Functional annotation and enrichment of DEGs in KEGG (Kyoto Encyclopedia of Genes and Genome; https://www.genome.jp/kegg) pathways was done as reported earlier (Deng et al., 2021).

### qRT-PCR Validation of Selected DEGs

We checked the expression of 17 DEGs through qRT-PCR analysis in order to validate the transcriptome results. The Actin gene was used as an internal control to standardize the expression results (Asif et al., 2014). The PCR reactions and subsequent analysis of the expression data were carried out as reported earlier (Deng et al., 2021) on a Rotor-Gene 6,000 machine (Qiagen, Shanghai, China) using primers designed in Primer 3 (http://frodo.wi.mit.edu/primer3/) (Table 2).

**TABLE 2:** List of primers used for qRT-PCR analysis of selected genes in *M. nana* peel treated with or without UV-C.

Gene Id	Primer forward sequence (5′-3′)	Primer reverse sequence (5′-3′)
*Ma01_g04420*	AGTGTTGGCTTTGTCTAT	CGGTTCCATTCTTCTT
*Ma05_g03720*	AAG​TAG​TCC​CAT​TTG​TTC​T	CAAGGGCTAAGGTTCA
*Ma10_g05180*	CAAGCATTGGGATTTT	GTTACCACGGACACGA
*Ma07_g23680*	GCCTAAAGGTCCTCTGT	GCTGTCCGTGTCAATC
*Ma03_g26620*	TCT​ATG​ACT​TGG​AGG​AAC​A	TGCTGGAACTACTGACG
*Ma07_g03980*	ATCCCTTGGCAGTAAA	ATTCGTAAACCCTCAG
*Ma07_g07210*	GCCCGTTCACCAAGTC	AAGCCCAAGAGCCAATG
*Ma03_g26150*	GGTACAATCGCTCCATC	GAACGCTTTCTCCTCA
*Ma11_g18320*	CAGGGTTTATGGAGGG	ACATTCTGGCTTTGGC
*Ma07_g11110*	TCCCTGTTTCCACCTC	TTATTTCCTCGGATTCTC
*Ma04_g24880*	CTGTCTCCCTTTCGTC	TGATGCCTCAGTGTTG
*Ma07_g18160*	GTGCGGCAGAAGTGTC	TTCTTGAATCGGGAGG
*Ma04_g39080*	ATG​GGT​TCA​AAA​ATC​TGC​TTC	TCA​ATT​AGA​ATT​GAC​CTT​CCT​G
*Ma05_g07440*	ATG​GCT​GCA​AGA​AAC​CCA​T	CTA​AGT​TTT​TGG​GTG​TTT​CCC
*Ma09_g10880*	ATG​ATG​TCA​TTA​ACC​GCC​GT	CTA​CAA​AAT​GGA​ACA​AGC​A
*Ma09_g30440*	ATG​GCC​GTA​CCT​TTC​GCC​AC	TTA​TCC​ACG​CAT​GGC​AAT​C
*Ma05_g00960*	ATG​GCT​CAA​TAC​GGG​AGG​CA	CTA​TGA​ATT​GGT​CTT​CCT​G
*Actin*	ATG​GGA​AAA​ACA​GAA​TTT​TG	TCA​TCT​ATT​GTT​AGA​CAG​A

### Metabolome Analysis

Sample preparation, extraction, UPLC analysis, and ESI-Q TRAP-MS/MS analysis of both UV-C treated banana peels and CK was done as reported earlier (Lee and Kim, 2013). All the statistical procedures and data analyses were performed as reported earlier (Deng et al., 2021).

### Co-Joint Analysis of Omics Data

Co-joint analyses of DEGs and DAMs was done to establish data relationships between different levels of transcripts and metabolites (Jozefczuk et al., 2010). First to determine the degree of enrichment of pathways. The DEGs and DAMs were mapped on the KEGG pathways at the same time and displayed as histogram. We used CCA package (González et al., 2008) in R to calculate the PCC of DEG and DAMs and a correlation coefficient cluster heatmap of DEGs and DAMs was drawn. Furthermore, the results of PCC were used to generate gene-metabolite networks.

## Abbreviations

DEG; differentially expressed gene, FPKM; fragments per kilobase of exon model per million reads mapped, FDR; false discovery rate, GO; gene ontology, KEGG; Kyoto Encyclopedia of Genes and Genomes, PCA; principal component analysis, qRT-PCR; quantitative real-time PCR, TF; transcription factor.

## Data Availability

The original contributions presented in the study are publicly available in NCBI under accession number PRJNA731314.

## Abbreviations

DEG; differentially expressed gene, FPKM; fragments per kilobase of exon model per million reads mapped, FDR; false discovery rate, GO; gene ontology, KEGG; Kyoto Encyclopedia of Genes and Genomes, PCA; principal component analysis, qRT-PCR; quantitative real-time PCR, TF; transcription factor.
