# Supplementary material for: Combined Transcriptome and Metabolome Analysis of Musa nana Laur. Peel Treated With UV-C Reveals the Involvement of Key Metabolic Pathways
Source: Front Genet. 2022 Jan 27;12:792991. doi: 10.3389/fgene.2021.792991 (PMC8830439; doi:10.3389/fgene.2021.792991)
Supplement: Supplementary file 1 [file DataSheet1.docx]

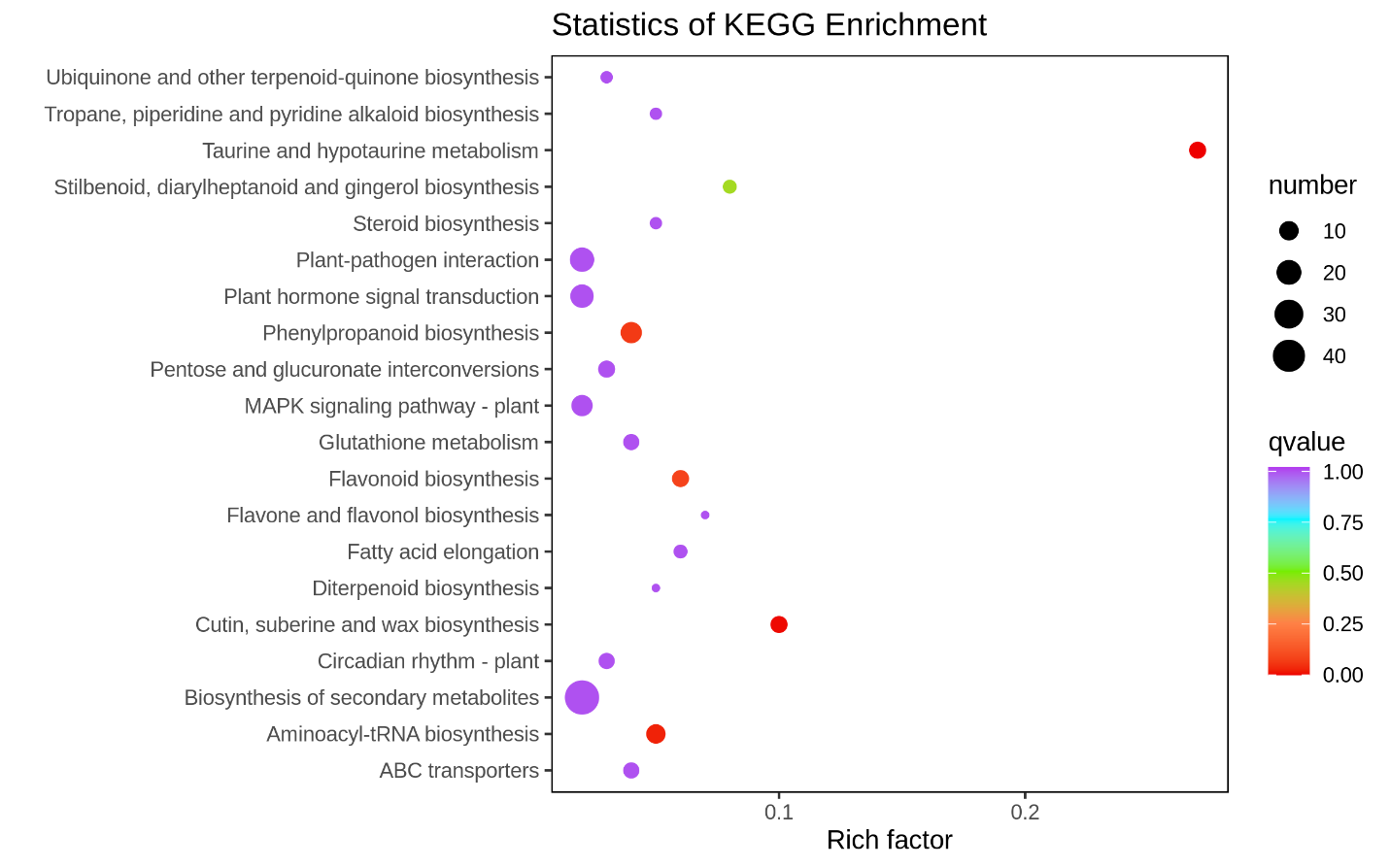


**Supplementary Figure 1.** Enrichment of differentially expressed genes between UV-C treated and CK *Musa nana* peels in different pathways.


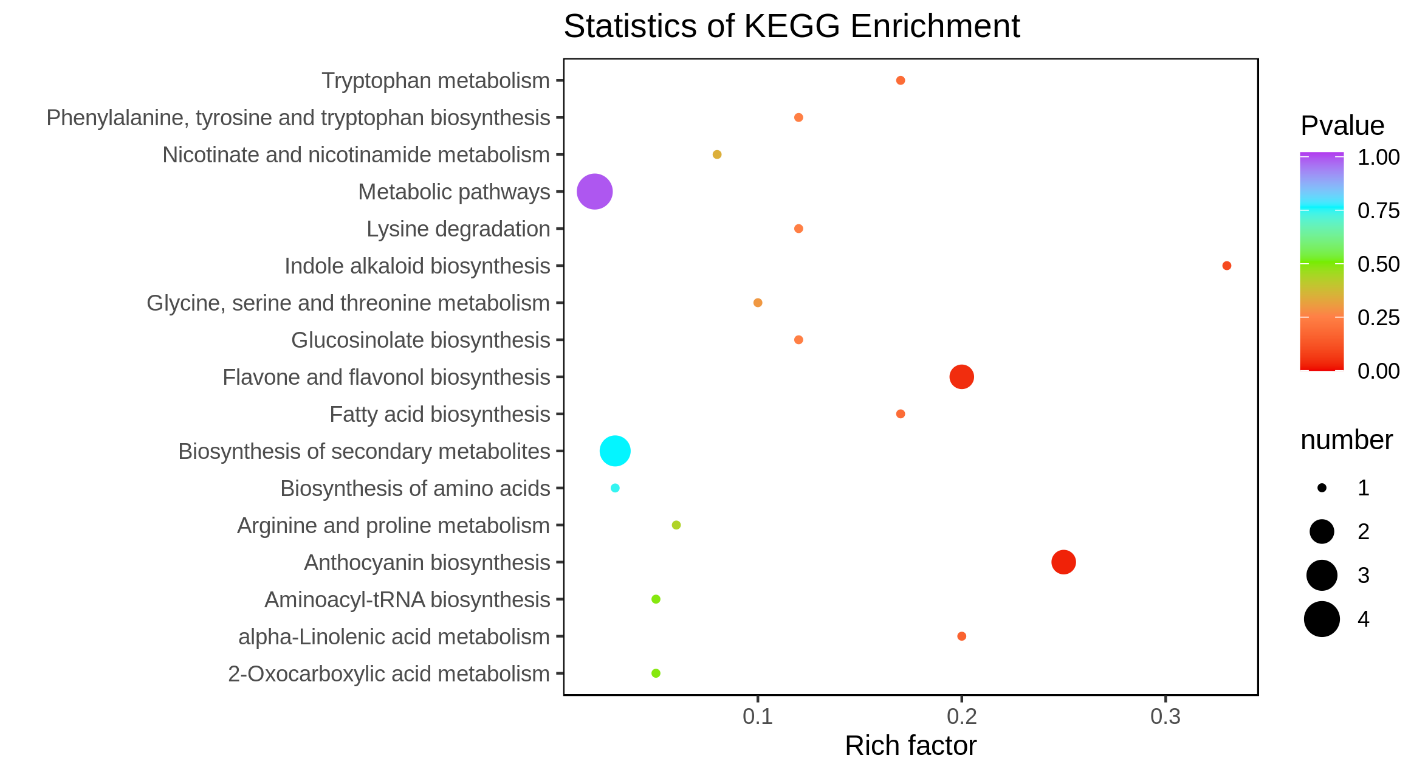


**Supplementary Figure 2.** Enrichment of differentially accumulated metabolites between UV-C treated and CK *Musa nana* peels in different pathways.
